# The association between intrapartum interventions and immediate and ongoing breastfeeding outcomes: an Australian retrospective population-based cohort study

**DOI:** 10.1186/s13006-022-00492-7

**Published:** 2022-07-05

**Authors:** Madison S. Andrew, Roshan J. Selvaratnam, Miranda Davies-Tuck, Kim Howland, Mary-Ann Davey

**Affiliations:** 1 Department of Obstetrics and Gynaecology, Monash University, Level 5, Monash Medical Centre, 246 Clayton Road, Clayton, VIC 3168 Australia; 2 The Ritchie Centre, The Hudson Institute, Clayton, Victoria Australia; 3 Municipal Association of Victoria, Melbourne, Victoria Australia

**Keywords:** Breast feeding, Analgesia, Epidural, Caesarean section, Synthetic oxytocin

## Abstract

**Background:**

The use of intrapartum interventions is becoming increasingly common globally. Interventions during birth, including caesarean section (CS), epidural analgesia and synthetic oxytocin infusion, can be important in optimizing obstetric care, but have the potential to impact breastfeeding. This study aimed to identify whether women who have certain intrapartum interventions have greater odds of unfavourable breastfeeding outcomes, both the immediate post-partum period and in the months after birth.

**Methods:**

This was a population-based cohort study of singleton livebirths at ≥37 weeks’ gestation between 2010 and 2018 in Victoria, Australia using routinely-collected state-wide data from the Victorian Perinatal Data Collection (VPDC) and the Child Development Information System (CDIS). The interventions included were pre-labour CS, in-labour CS, epidural analgesia, and synthetic oxytocin infusion (augmentation and/or induction of labour). Outcomes were formula supplementation in hospital, method of last feed before hospital discharge and breastfeeding status at 3-months and 6-months. Descriptive statistics and multivariable logistic regression models adjusting for potential confounders were employed.

**Results:**

In total, 599,191 women initiated breastfeeding. In-labour CS (aOR 1.96, 95%CI 1.93,1.99), pre-labour CS (aOR 1.75, 95%CI 1.72,1.77), epidural analgesia (aOR 1.45, 95%CI 1.43,1.47) and synthetic oxytocin infusion (aOR 1.24, 95%CI 1.22,1.26) increased the odds of formula supplementation in hospital. Long-term breastfeeding data was available for 105,599 infants. In-labour CS (aOR 0.79, 95%CI 0.76,0.83), pre-labour CS (aOR 0.73, 95%CI 0.71,0.76), epidural analgesia (aOR 0.77, 95%CI 0.75,0.80) and synthetic oxytocin infusion (aOR 0.89, 95%CI 0.86–0.92) decreased the odds of exclusive breastfeeding at 3-months post-partum, which was similar at 6-months. There was a dose-response effect between number of interventions received and odds of each unfavourable breastfeeding outcome.

**Conclusion:**

Common intrapartum interventions are associated with less favourable breastfeeding outcomes, both in hospital and in the months after birth. This confirms the importance of only undertaking interventions when necessary. When interventions are used intrapartum, an assessment and identification of women at increased risk of early discontinuation of breastfeeding has to be performed. Targeted breastfeeding support for women who have intrapartum interventions, when they wish to breastfeed, is important.

## Background

Despite more than 95% of women initiating breastfeeding after birth in Australia [1], early discontinuation and supplementation with infant formula, a known influence on long-term breastfeeding success [2]. is common [3] At 6 months of age, fewer than 15% of infants are exclusively breastfeeding and only half of infants receive any breastmilk [4]. The likelihood that a mother will breastfeed after birth, and sustain that breastfeeding until her child is at least 6 months of age, is influenced by a number of complex factors including maternal sociodemographic characteristics, professional and peer support networks, physiological state and intention to breastfeed [5–7]. There are several known barriers to successful breastfeeding including smoking, obesity, diabetes, multiple births and pre-term births. One factor that has garnered attention in recent years is the influence of perinatal interventions. Intrapartum interventions, specifically caesarean section (CS), epidural analgesia, and synthetic oxytocin infusion, have been proposed to interfere with optimal breastfeeding success through various mechanisms. For example, reduced skin-to-skin contact after CS has been shown to affect lactation by limiting infant pre-feeding behaviours, maternal-infant bonding and maternal oxytocin release [8]. Skin-to-skin contact, which involves positioning the dried, naked infant prone on the mother’s bare chest, and zero separation between the newborn baby and the mother immediately after birth, are well-known protective factors for breastfeeding establishment [9] particularly after CS [10, 11]. Opioid analgesia, such as those in epidurals, can cross the blood brain barrier in the fetus and affect feeding reflexes [12]. Additionally, it is hypothesised that oxytocin infusions desensitise oxytocin receptors in the breast, weakening the milk-ejection reflex [13].

Australia, similar to other high-income countries, has seen rapid rises in rates of intervention use [14]. In Victoria in 2019, 37.2% of women had a CS, 40.1% used epidural analgesia and 46.6% had labour either induced or augmented, rates that have significantly increased from previous decades [15]. So far, studies assessing the impact of such birth interventions on breastfeeding show mixed results. Though various studies have associated these interventions with unfavourable early breastfeeding outcomes, few explore the ongoing impact on breastfeeding. In particular, the impact of receiving multiple interventions on breastfeeding outcomes in hospital is poorly considered. Identification of women at increased risk of early discontinuation of breastfeeding would enable provision of targeted breastfeeding supports for these women so that the benefits of breastfeeding are not lost to the increasing iatrogenesis in maternity care.

In this study, we aimed to explore the association between CS, epidural analgesia, and synthetic oxytocin infusion, both individually and when used in combination, and short and longer-term breastfeeding outcomes.

## Methods

### Data sources and study population

This retrospective population-based cohort study used deidentified data from the Victorian Perinatal Data Collection (VPDC), a validated dataset [16] of routinely collected state-wide information for every birth in Victoria, Australia, of ≥20 weeks’ gestation (or ≥ 400 g birth weight if gestation is unknown). Variables include mode of birth, type of intrapartum analgesia, use of oxytocin infusions in labour, initiation of breastfeeding, use of infant formula in hospital and feeding status at discharge from hospital. We analysed all singleton term livebirths at ≥37 weeks gestation whose mother initiated breastfeeding in hospital between 1 January 2010 and 31 December 2018. Breastfeeding initiation refers to whether the baby was put to the breast at least once or whether any attempt was made to express breastmilk during the hospital stay.

A subset of this population, namely those term singleton livebirths born between 1 January 2015 and 31 December 2017, were linked to the Child Development Information System (CDIS) containing information on infant feeding status at 3-month and 6-months. These dates were chosen because of the availability of linked data within this timeframe. The CDIS is a centralised dataset reporting ongoing breastfeeding outcomes through the Maternal and Child Health (MCH) service. The MCH service is a universal primary health service that provides all Victorian families with 10 consultations with MCH nurses between birth and school age, with additional as required. At each consultation parents report their child’s feeding status as exclusively breastfeed, partially breastfed or formula-fed. The most recent total participation rates for the MCH service were shown to be 94.1% at 4-months and 85.8% at 8-months [17], when parents report their child’s feeding status at 3-months and 6-months, respectively. Almost all Local Government Areas reported their MCH information to CDIS by the study period, 2015–17, enabling linkage with births in 2015–17. The linkage was conducted by the Centre for Victorian Data Linkage based on a concordance of predetermined identifiers.

### Outcomes, exposures and covariates

The primary short-term breastfeeding outcomes were formula supplementation in hospital, defined as breast-fed babies receiving any amount of formula during the initial hospital stay, and last feed at the breast, defined as having the last feed before hospital discharge directly and exclusively from the breast, and therefore excludes expressed breast milk. These were assessed using the total study population.

The primary long-term breastfeeding outcomes were breastfeeding status at 3-months and 6-months of age, which was recorded as either exclusive, any or no breastfeeding. Exclusive breastfeeding is defined as receiving only breastmilk as the source of nutrition and includes expressed breast milk. Any breastfeeding includes receiving any breastmilk, in addition to artificial milk sources, water-based drinks or solid food. Long-term breastfeeding outcomes were assessed using the linked VPDC-CDIS dataset sub-study population for births in 2015–17.

The exposure variables were four intrapartum interventions: pre-labour CS, in-labour CS, epidural analgesia, and synthetic oxytocin infusion. Method of birth was categorised as either pre-labour CS, in-labour CS, or vaginal birth. Epidural analgesia included epidural block, spinal block and combined spinal/epidural block used to relieve pain in labour. We did not assess epidural anaesthesia provided de novo to facilitate operative birth because of the brief fetal exposure. Synthetic oxytocin infusion includes that which was used for induction and/or augmentation of labour.

Several covariates were assessed. These included socioeconomic status, parity (primiparous, multiparous), hospital admissions status (public, private), birthweight (< 2500 g, 2500-3999 g, ≥4000 g), maternal region of birth, maternal age, maternal body mass index (BMI), smoking status during pregnancy, marital status (married, de facto, single), and gestation at birth. Socioeconomic status was defined using the Socio-Economic Indexes for Areas (SEIFA) indices [18]. The indices are based on information from the five-yearly Census of Population and Housing and are divided into quintiles, with 1 being the most disadvantaged and 5 being the least disadvantaged. The SEIFA used here is based on the smallest available residential neighbourhood level (SA1). Maternal region of birth was classified by the Standard Australian Classification of Countries (SACC) [19]. Marital status information was available for the total population but not for the linked VPDC-CDIS sub-population.

### Analyses

We first described the rates of intrapartum interventions and breastfeeding outcomes in our populations. Categorical variables were reported as absolute numbers and percentages and compared using Pearson’s Chi-square test. To assess the association between each intervention and breastfeeding outcomes, we then performed univariable and multivariable logistic regression models to obtain adjusted odds ratios (aOR) with 95% confidence intervals (CI), adjusting for available confounders. Given that the use of each intervention was not always mutually exclusive, each intrapartum intervention was first assessed regardless of whether other interventions were also used, then each was assessed when used in isolation, and finally cumulatively. A two-tailed *P*-value of < 0.05 was deemed statistically significant.

Missing data were excluded case-wise. Data were missing for 8676 (1.4%) cases regarding formula supplementation in hospital, 3808 (0.6%) cases regarding last feed at breast and 41 cases (< 0.1%) regarding method of birth. There was no missing data on epidural analgesia and synthetic oxytocin infusion. Data were missing for < 0.1% of mothers regarding parity, maternal age, sex of baby and hospital admission status, 53,029 (8.9%) cases regarding smoking status, 39,181 (6.5%) cases regarding maternal BMI, 7744 (1.3%) cases regarding marital status, 37,224 (6.2%) cases regarding SEIFA quintile, and 3681 (0.6%) cases regarding country of birth region. Within the linked population, feeding status information was missing for 16,269 (15.4%) of infants at 3-months and 10,072 (9.5%) of infants at 6-months. A ‘missing’ category was created for covariates with substantial missing data in order to include cases with valid data on other variables in multivariable analyses.

Statistical analyses were performed using Statistical Package for the Social Science Version 26 (SPSS, IBM Corp., Armonk, New York, USA) and Stata SE Version 16 (Stata, 2020, release 16, StataCorp, Texas, USA).

## Results

Breastfeeding was initiated by 95.1% (*n* = 599,191) of all singleton livebirths at ≥37 weeks gestation in hospital between 2010 and 2018. Thus, our total study population was *n* = 599,191Of these, 197,775 infants were born between 2015 and 2017, of which 105,599 (53.4%) of infants had VPDC data that linked to long-term feeding status in the CDIS dataset. The characteristics of the total population and the restricted population with linked long-term breastfeeding outcomes were largely similar, though infants in the linked population were more likely to be born at 37 weeks and in a private hospital, mothers were more likely to have a higher SES and less likely to have smoked in pregnancy (Table 1).

**Table 1:** Maternal and infant characteristics of the population

	All births (*N* = 599,191)^a^	Births that linked to CDIS dataset (*N* = 105,599)^a^
	N (%)	N (%)
Parity		
Primiparous	269,018 (44.9)	48,526 (46.0)
Multiparous	330,132 (55.1)	57,073 (54.0)
Sex of Baby		
Male	306,177 (51.1)	54,184 (51.3)
Female	292,821 (48.9)	51,415 (48.7)
Admission Type		
Public hospital	444,742 (74.2)	74,781 (70.8)
Private hospital	154,449 (25.8)	30,797 (29.2)
Maternal Age Group		
< 25 years	69,222 (11.6)	9301 (8.8)
25–29 years	157,697 (26.3)	26,426 (25.0)
30–34 years	220,975 (36.9)	41,845 (39.6)
35–39 years	123,308 (20.6)	23,124 (21.9)
40 years or older	27,800 (4.6)	4903 (4.6)
BMI		
Mean (SD)	25.78 (5.79)	25.58 (5.62)
Marital Status		
Married	424,745 (71.8)	NA
De facto	102,758 (17.4)	NA
Single	63,944 (10.8)	NA
Gestation (completed weeks)		
37	48,828 (4.1)	9161 (8.7)
38	138,038 (23.0)	25,157 (23.8)
39	175,726 (29.3)	32,090 (30.4)
40	156,018 (26.0)	27,201 (25.8)
41	76,262 (12.7)	11,561 (10.9)
42 or more	4319 (0.7)	425 (0.4)
SEIFA quintile		
1 (most disadvantaged)	109,356 (19.5)	17,513 (16.7)
2	111,313 (19.8)	20,151 (19.3)
3	113,055 (20.1)	21,927 (21.0)
4	113,558 (20.2)	21,824 (20.9)
5 (least disadvantaged)	114,685 (20.4)	23,206 (22.2)
Smoking status during pregnancy		
Yes	53,029 (9.2)	7038 (7.0)
Country of Birth Region		
Australia	378,658 (63.6)	69,426 (66.1)
Oceania and Antarctica	16,610 (2.8)	2258 (2.1)
Americas	8338 (1.4)	1244 (1.2)
South-East Asia	38,698 (6.5)	6148 (5.8)
Southern and Central Asia	60,621 (10.2)	9827 (9.3)
North-East Asia	29,507 (5.0)	6220 (5.9)
North-West Europe	17,760 (3.0)	3280 (3.1)
Southern and Eastern Europe	11,524 (1.9)	1676 (1.6)
North Africa and the Middle East	20,718 (3.5)	3125 (3.0)
Sub-Saharan Africa	13,076 (2.2)	1906 (1.8)

*Abbreviation*: *BMI* body mass index, *SEIFA* Socio-Economic Indexes for Areas, *NA* not available

^a^Missing values are excluded from the denominator

Within the total birth population, 19.1% (*n* = 114,251) had a pre-labour CS, 12.8% (*n* = 76,516) had an in-labour CS, 26.1% (*n* = 156,395) had epidural analgesia and 31.0% (*n* = 185,628) had synthetic oxytocin infusion (augmentation and/or induction of labour). Overall, 38.0% (*n* = 227,499) had none of the assessed interventions, 40.4% had one (*n* = 242,083), 16.4% had two (*n* = 98,158) and 5.2% (*n* = 31,451) had all three intrapartum interventions. The demographic information of women within each intervention group is presented in Table 2. Intervention use was more common among primiparous women, with the exception of pre-labour CS, where significantly more women were multiparous compared to primiparous (72.2% vs 27.8%, *P* < 0.001). Intervention use was also more common with increasing SEIFA quintile, at private hospitals and with increasing maternal BMI (all *P* < 0.001). Infant gestational age and birth weight was relatively similar for all groups, except for pre-labour CS, where infants were born approximately 1 week earlier than those born by vaginal birth or in-labour CS.

**Table 2:** Maternal and infant characteristics of all births, by intrapartum intervention

	Method of Birth			Epidural Analgesia		Synthetic oxytocin infusion	
	Vaginal birth (*n* = 408,383)	Pre-labour CS (*n* = 114,251)	In-labour CS (*n* = 76,516)	No (*n* = 442,796)	Yes (*n* = 156,395)	No (*n* = 413,563)	Yes (*n* = 185,628)
Parity							
Primiparous	180,921 (44.3)	31,813 (27.8)	56,259 (73.5)	160,620 (36.3)	108,398 (69.3)	147,368 (35.6)	121,650 (64.4)
Multiparous	227,433 (55.7)	82,431 (72.2)	20,252 (26.5)	282,143 (69.7)	47,989 (30.7)	266,170 (65.5)	63,962 (34.5)
Admission Type							
Public	317,464 (77.7)	71,240 (62.4)	56,030 (73.2)	339,853 (76.8)	104,889 (67.1)	308,281 (74.5)	136,461 (73.5)
Private	90,919 (22.3)	43,011 (37.6)	20,486 (26.8)	102,943 (23.2)	51,506 (32.9)	105,282(25.5)	49,167 (26.5)
Maternal age (years), mean (SD)							
	30.41 (5.18)	32.99 (4.96)	30.78 (5.08)	31.10 (5.28)	30.54 (5.05)	31.15 (5.24)	30.51 (5.16)
Maternal BMI, mean (SD)							
	25.31 (23.45)	27.23 (31.24)	26.49 (6.08)	25.82 (22.67)	25.84 (26.73)	25.73 (28.32)	26.06 (5.99)
Marital Status							
Married	284,337 (66.9)	87,479 (77.3)	52,897 (70.0)	315,540 (72.2)	109,205 (70.7)	296,453 (72.6)	128,292 (70.0)
De Facto	71,668 (17.8)	17,144 (15.2)	13,938 (18.5)	74,858 (17.1)	27,900 (18.1)	69,219 (17.0)	33,539 (18.3)
Single	46,784 (11.6)	8479 (7.5)	8690 (11.5)	46,560 (10.7)	17,384 (11.3)	42,528 (10.4)	21,416 (11.7)
Gestation (completed weeks), mean (SD)							
	39.27 (1.15)	38.49 (0.94)	39.43 (1.25)	39.06 (1.15)	39.37 (1.19)	39.08 (1.13)	39.30 (1.26)
Birth Weight (grams), mean (SD)							
	3450 (488)	3413 (507)	3510 (515)	3443 (498)	3474 (489)	3455 (492)	3442 (505)
SEIFA quintile							
1 more disadvantaged	77,744 (20.3)	17,496 (16.3)	14,111 (19.7)	85,568 (20.6)	23,788 (16.2)	76,129 (19.7)	33,227 (19.0)
2	76,561 (20.0)	20,073 (18.7)	14,673 (20.5)	83,648 (20.2)	27,665 (18.8)	76,731 (19.8)	34,582 (19.8)
3	76,745 (20.1)	21,636 (20.1)	14,667 (20.5)	83,361 (20.1)	29,694 (20.2)	77,844 (20.1)	35,211 (20.1)
4	75,861 (19.8)	23,237 (21.6)	14,451 (20.2)	81,971 (19.8)	31,587 (21.5)	77,824 (20.1)	35,734 (20.4)
5 least disadvantaged	75,829 (19.8)	25,101 (23.3)	13,742 (19.2)	80,173 (19.3)	34,512 (23.4)	78,548 (20.3)	36,137 (20.7)
Smoking status during pregnancy							
Yes	37,890 (9.6)^a^	8345 (7.7)	6790 (9.3)^a^	39,256 (9.2)^b^	13,773 (9.3)^b^	36,008 (9.1)	17,021 (9.5)

*Abbreviation*: *BMI* body mass index, *SEIFA* Socio-Economic Indexes for Areas, *CS* caesarean section

Data given as number (proportion %) unless otherwise specified. Proportions reported as column percentages

*P*-value for differences in characteristics between methods of birth, use of epidural analgesia and synthetic oxytocin infusion < 0.001 unless otherwise specified

^a^*P*-value for smoking status during pregnancy between vaginal birth and in-labour CS = 0.011

^b^*P*-value for smoking status during pregnancy between no epidural analgesia and yes epidural analgesia = 0.709

Formula supplementation was used by 28.2% (*n* = 166,642) of infants in hospital and 78.2% (*n* = 465,695) of infants had their last feed at the breast. The proportion of infants who had formula in hospital was significantly higher following in-labour CS and pre-labour CS compared to vaginal birth (41.7 and 38.0% vs 23.0%, both *P* < 0.001), following epidural compared to no epidural (35.4% vs 25.7%, *P* < 0.001) and following oxytocin infusion compared to no oxytocin infusion (33.3% vs 25.9%, *P* < 0.001) (Table 3). As the number of interventions received increased, the proportion of infants who received formula in hospital increased, from 18.0% for women who had none of the assessed interventions to 45.7% for women who had all three interventions (*P* < 0.001). The inverse was true for receiving the last feed before discharge directly at the breast.

**Table 3:** Adjusted multinomial logistic regression, associations between intrapartum interventions and breastfeeding outcomes in hospital

	Formula Given in Hospital			Last Feed at Breast		
	N (%)	Adjusted OR (95% CI)^a^	*P* value	N (%)	Adjusted OR (95% CI)	*P value*
Method of Birth						
Vaginal Birth	92,467 (23.0)	1	–	330,867 (81.5)	1	–
Pre-labour CS	42,667 (38.0)	1.75 (1.72–1.77)	< 0.001	81,799 (72.1)	0.66 (0.65–0.67)	< 0.001
In-labour CS	31,488 (41.7)	1.96 (1.93–1.99)	< 0.001	53,001 (69.7)	0.65 (0.64–0.66)	< 0.001
Epidural Analgesia						
No	112,057 (25.7)	1	–	353,235 (80.3)	1	–
Yes	54,585 (35.4)	1.45 (1.43–1.47)	< 0.001	112,460 (72.4)	0.71 (0.70–0.72)	< 0.001
Synthetic Oxytocin Infusion						
No	105,774 (25.9)	1	–	330,523 (80.4)	1	–
Yes	60,868 (33.3)	1.24 (1.22–1.26)	< 0.001	135,172 (73.3)	0.78 (0.77–0.79)	< 0.001
Mutually Exclusive Interventions						
No Intervention	40,360 (18.0)	1	–	193,968 (85.7)	1	0
Pre-labour CS only	41,333 (37.7)	2.17 (2.13–2.21)	< 0.001	80,226 (72.5)	0.53 (0.52–0.54)	< 0.001
In-labour CS only	7011 (37.0)	2.17 (2.10–2.24)	< 0.001	14,178 (74.3)	0.59 (0.57–0.61)	< 0.001
Epidural analgesia only	11,048 (28.3)	1.63 (1.59–1.67)	< 0.001	30,834 (78.2)	0.64 (0.62–0.66)	< 0.001
Synthetic oxytocin infusion only	17,744 (25.1)	1.30 (1.27–1.33)	< 0.001	56,834 (79.2)	0.74 (0.73–0.76)	< 0.001
Number of Interventions Received						
None	40,360 (18.0)	1	–	193,968 (85.7)	1	–
One	77,136 (32.4)	1.77 (1.74–1.79)	< 0.001	181,612 (75.5)	0.61 (0.60–0.62)	< 0.001
Two	34,989 (36.2)	2.08 (2.04–2.12)	< 0.001	69,547 (71.4)	0.52 (0.51–0.53)	< 0.001
Three	14,157 (45.7)	2.90 (2.83–2.98)	< 0.001	20,568 (65.8)	0.43 (0.42–0.45)	< 0.001

*Abbreviation*: *OR* odds ratio, *CI* confidence interval, *CS* caesarean section

^a^Adjusted for parity, maternal BMI, sex of baby, smoking status during pregnancy, SEIFA quintile, infant birth weight, hospital admission status, infant gestational age, country region of birth, marital status and maternal age

In adjusted analyses, all interventions were significantly associated with less favourable breastfeeding outcomes in hospital, including when separated into mutually exclusive groups (Table 3). In-labour CS was the strongest predictor of formula supplementation in hospital (aOR 1.96, 95% CI 1.93–1.99), followed by pre-labour CS (aOR 1.75, 95% CI 1.72–1.77), epidural analgesia (aOR 1.45, 95% CI 1.43–1.47) and then oxytocin infusion (aOR 1.24, 95% CI 1.22–1.26). When performed in isolation, both pre-labour CS and in-labour CS equally predicted formula supplementation (aOR 2.17, 95% CI 2.13–2.21 and aOR 2.17, 95% CI 2.10–2.24). There was a dose response relationship between the number of interventions received and formula supplementation in hospital. Compared to no interventions, having one (aOR 1.77, 95% CI 1.74–1.79), two (aOR 2.08, 95% CI 2.04–2.12) or all three (aOR 2.90 95% CI 2.83–2.98) interventions significantly increased the odds of formula supplementation in hospital. The inverse was true for receiving the last feed before discharge directly at the breast.

In the linked population, 52.4% (*n* = 46,703) and 14.5% (*n* = 13,713) were exclusively breastfeeding at 3-months and 6-months, respectively, and 70.8% (*n* = 63,088) and 55.4% (*n* = 52,400) of mothers were breastfeeding at all at 3-months and 6-months, respectively. Exclusive and any breastfeeding were less common at each time point for those who received each intervention compared to those who did not. After adjusting for confounders, each intervention remained associated with less favourable breastfeeding outcomes at 3-months and 6-months, and this persisted when the interventions were explored as mutually exclusive groups (Table 4a, b). The association was strongest for women who had pre-labour CS, which decreased the odds of exclusive and any breastfeeding at 3 months (aOR 0.73, 95% CI 0.71–0.76 and aOR 0.70, 95% CI 0.68–0.73, respectively); and of exclusive and any breastfeeding at 6 months (aOR 0.81, 95% CI 0.77–0.86 and aOR 0.72, 95% CI 0.69–0.74, respectively). There was a dose-response relationship between number of interventions received and the adjusted odds of exclusive and any breastfeeding at 3-months and 6-months.

**Table 4:** Adjusted multivariable logistic regression, associations between intrapartum interventions and breastfeeding outcomes at 3-months and 6-months post-partum

	Exclusive breastfeeding at 3-months			Any breastfeeding at 3-months			Exclusive breastfeeding at 6-months			Any breastfeeding at 6-months		
	N (%)	AdjOR^a^ (95% CI)	*P*-value	N (%)	AdjOR^a^ (95% CI)	*P*-value	N (%)	AdjOR^a^ (95% CI)	*P*-value	N (%)	AdjOR^a^ (95% CI)	*P*-value
Method of Birth												
Vaginal Birth	32,636 (54.9)	1	–	43,211 (72.7)	1	–	9622 (15.2)	1	–	36,446 (57.7)	1	–
Pre-labour CS	8789 (47.9)	0.73 (0.71–0.76)	< 0.001	12,308 (67.1)	0.70 (0.68–0.73)	< 0.001	2512 (13.0)	0.81 (0.77–0.86)	< 0.001	9781 (50.6)	0.72 (0.69–0.74)	< 0.001
In-labour CS	5278 (46.9)	0.79 (0.76–0.83)	< 0.001	7569 (69.2)	0.79 (0.75–0.83)	< 0.001	1579 (13.1)	0.88 (0.82–0.93)	< 0.001	6172 (51.2)	0.80 (0.77–0.84)	< 0.001
Epidural Analgesia												
No	34,776 (54.3)	1	–	46,289 (72.3)	1	–	10,166 (15.0)	1	–	38,949 (57.4)	1	–
Yes	11,927 (47.6)	0.77 (0.75–0.80)	< 0.001	16,799 (67.1)	0.77 (0.74–0.80)	< 0.001	3547 (13.3)	0.89 (0.85–0.93)	< 0.001	13,451 (50.5)	0.76 (0.74–0.78)	< 0.001
Synthetic Oxytocin Infusion												
No	32,052 (54.2)	1	–	42,607 (72.1)	1	–	9429 (15.0)	1	–	35,711 (56.8)	1	–
Yes	14,651 (48.9)	0.89 (0.86–0.92)	< 0.001	20,481 (68.4)	0.91 (0.88–0.94)	< 0.001	4284 (13.5)	0.95 (0.91–0.99)	< 0.001	16,689 (52.8)	0.92 (0.90–0.95)	< 0.001
Number of Interventions Received												
None	18,014 (59.2)	1	–	23,096 (75.9)	1	–	5361 (16.6)	1	–	20,073 (62.0)	1	–
One	18,935 (50.2)	0.71 (0.68–0.73)	< 0.001	26,070 (69.1)	0.70 (0.66–0.72)	< 0.001	5431 (13.6)	0.82 (0.79–0.86)	< 0.001	21,143 (53.0)	0.69 (0.67–0.72)	< 0.001
Two	7524 (47.6)	0.65 (0.63–0.68)	< 0.001	10,642 (67.3)	0.65 (0.62–0.68)	< 0.001	2258 (13.4)	0.82 (0.78–0.87)	< 0.001	8570 (51.0)	0.66 (0.63–0.69)	< 0.001
Three	2197 (43.7)	0.60 (0.56–0.64)	< 0.001	3236 (64.3)	0.58 (0.53–0.62)	< 0.001	648 (12.2)	0.75 (0.68–0.83)	< 0.001	2576 (48.4)	0.61 (0.57–0.65)	< 0.001

*Abbreviations: OR* odds ratio, *CI* confidence interval

^a^ Adjusted for parity, maternal BMI, SEIFA quintile, smoking status during pregnancy, hospital admission status, maternal age, maternal country region of birth, and infant birth weight

## Discussion

In this study we demonstrated that the use of each of the intrapartum interventions investigated was associated with less favourable breastfeeding outcomes, both in hospital and in the months after birth. The associations persisted when each intervention was assessed independently from other interventions in mutually exclusive groups. In-labour CS was the strongest predictor of formula supplementation in hospital, though pre-labour CS was the strongest predictor of not breastfeeding at 3 and 6-months. Importantly, the associations increased in strength with increasing number of interventions received in a dose-response fashion. These findings suggest the need for targeted breastfeeding support in hospital and after discharge for women who receive these intrapartum interventions.

The use of intrapartum interventions was common in our study, with nearly two thirds of women receiving at least one intervention. This was not surprising given rates of intrapartum intervention use have rapidly increased in Australia over the last decade [15]. This highlights the need for greater discussion about limiting intervention use when not medically indicated or necessary. Moreover, women should be well-informed about the potential risks vs benefits of having an intervention to aid in shared and informed decision-making during birth. Alongside this, despite all women in our study population initiating breastfeeding, rates of formula supplementation in hospital were high and discontinuation of exclusive and any breastfeeding in the months after birth was common. Only one in two infants were exclusively breastfeeding at 3-months, and this decreased to one in seven infants at 6-months. These rates fall far short of the national targets for breastfeeding, such as those produced by the Australian National Breastfeeding Strategy, which aim for 40% of infants to be exclusively breastfeeding until around 6 months of age by 2022 [20] and international targets set by the World Health Organisation for at least 50% of infants to be exclusively breastfeeding to 6 months by 2025 [21].

The finding that each intrapartum intervention increased the likelihood of formula supplementation in hospital and decreased the likelihood of exclusive breastfeeding at discharge is in keeping with the results of several studies [22–24]. Novel to our findings, however, is that even when used in isolation, each intervention still negatively affected early breastfeeding outcomes. The underlying explanation for these findings is likely to be multifactorial. In respect to pre-labour CS, it has been suggested that the absence of labour may interfere with normal hormonal changes required for successful lactation [25]. However we observed that in-labour CS was a stronger predictor of less favourable early breastfeeding outcomes than pre-labour CS. This suggests that the stress and pain associated with operative birth also plays a role, possibly related to post-partum opiate pain relief, reduced skin-to-skin contact and poor mobility [26]. Physiologically, both fentanyl in epidural analgesia and synthetic oxytocin have the potential to transfer to the fetal circulation and depress newborn feeding behaviours, [12, 27] which is a key reason for formula supplementation in hospital [2]. Each intervention has also been shown to interfere with the normal production of lactation hormones, oxytocin and prolactin [28]. Despite these proposed mechanisms, some studies have found no association between each intervention and early breastfeeding outcomes [29–31]. Interestingly, these studies tend to be conducted in hospitals accredited under the Baby Friendly Hospital Initiative, a program that encourages breastfeeding [32], and include populations of women who have strong intentions to breastfeed. Our findings recommend the need for enhanced breastfeeding support following intervention use for women birthing in hospitals without such initiatives and facilitators.

Importantly, we also found that each intervention decreased the odds of exclusive and any breastfeeding at 3-months and 6-months when compared to no intervention. Of note, associations between each intervention and exclusive breastfeeding at 6-month were weak compared to other long-term breastfeeding outcomes. This may be because many infants commence solid food introduction by 6-months of age. Exclusive breastfeeding at 6-months of age is therefore not necessarily the most appropriate indicator of ongoing breastfeeding success in the local setting. While many studies have associated intervention use with reduced long-term breastfeeding success [22, 24, 33], others have contrastingly found no impact [29, 34, 35]. The complex nature of ongoing feeding choices likely explains such varied findings; attitudes towards breastfeeding and intervention use differ significantly across the globe, as does the availability of ongoing supports and return-to-work policies. Our findings of reduced long-term breastfeeding in women who underwent interventions are unlikely to be entirely a direct physiological consequence of the interventions themselves. Rather, they will be explained, at least in part, by the well-known associations between successful establishment of breastfeeding and long-term breastfeeding maintenance [2]. In particular, women who successfully initiate breastfeeding immediately after birth are more likely to sustain that breastfeeding when their child is 6-months old [2]. This highlights the importance of optimising early and ongoing breastfeeding support for mothers who have multiple interventions given their long-term association with breastfeeding success. Such supports may include increased uptake of the Baby Friendly Hospital Initiative in hospitals around Australia and improved access to professional and lay lactation support, such as MCH nurses, lactation consultants, the Australian Breastfeeding Association and peer-support [7].

To our knowledge, our study is the first to demonstrate a dose-response relationship between increasing numbers of interventions used and the odds of receiving formula in hospital. This relationship persisted for breastfeeding outcomes in the months after birth, where increasing numbers of intrapartum interventions were associated with decreased odds of both exclusive and any breastfeeding at 3-months and 6-months, which is consistent with other studies [36, 37]. These results are important because intervention use often occurs in a cascade, with more than one in five women in the present study receiving multiple interventions during birth. The dose-response relationship may be explained by amplification of the physiological burden when interventions are used in combination, the fact that women who have multiple interventions are more likely to have complicated births, and that women who opt for intervention use, at least when done so electively, may be less concerned about having “unnatural” feeding sources.

This study is strengthened by its use of linked population-level data that has strong statistical power and is representative of all births in Victoria, Australia, eliminating selection bias. The VPDC dataset has been reported as having high accuracy in internal validation studies [16]. There are important limitations. Firstly, the CDIS dataset has not had any validation studies. Second, as an observational study, we were not able to adjust for all potential confounders of breastfeeding success. One important confounder that was not accounted for was return to work commitments for women, which is known to influence long-term infant feeding choices [38–40]. We also had no information about previous breastfeeding experience, indication for the intrapartum interventions or indication for formula supplementation in hospital, all of which may influence feeding methods in hospital and after discharge. We did not have access to data about admission to special care nursey or neonatal intensive care unit after birth. Nonetheless, we know that factors such as Apgar score and SCN/NICU admission likely lie on the causal pathway between intervention use and breastfeeding outcome. While only half of infants in the sub-study population had information regarding long-term feeding status, this was not unexpected. Some families do not attend the MCH consultations where feeding status is obtained, either because infants born in Victoria live interstate or have moved elsewhere and are not eligible to receive MCH consultations, or because parents choose not to participate. The movement of MCH data from state Local Government Areas to the centralised CDIS database has been gradual, and between 2015 and 2017, several Local Government Areas had not reported MCH data to the centralised database. Some of the infants born between 2015 and 2017 also would not have reached 4 or 8 months by the end of data collection and so feeding status for these time points would not have been available. Nonetheless, our restricted linked population was shown to be similar in many characteristics as the total Victorian infant population, indicating that the sub-study population can be considered representative of the total birth population Due to the exclusion of pre-term births and multiple births, the findings of our study are not generalisable to these groups. Future studies that examine the impact of intrapartum interventions on primiparous women, pre-term births and multiple births, would further the understanding of this topic.

## Conclusion

Women who have intrapartum interventions, particularly multiple interventions, are at increased risk of less favourable early and long-term breastfeeding outcomes, and therefore should be carefully considered for additional breastfeeding support, if they wish to breastfeed. Limiting intrapartum interventions when not medically indicated or necessary while ensuring women remain informed about potential risks is essential.

## Data Availability

This study is based on Victorian population perinatal and developmental data. These data do not belong to the authors but to the Consultative Council on Obstetric and Paediatric Mortality and Morbidity and the Municipal Association of Victoria, respectively. The authors are not permitted to share them, except in aggregate (as, for example, in a publication). However, interested parties can obtain the deidentified data on which this study is based by submitting a research protocol to the Victorian Agency for Health Information Data Request Hub at: https://vahi.freshdesk.com/support/home.

## Abbreviations

aOR: Adjusted Odds Ratio
CCOPMM: Consultative Council on Obstetric and Paediatric Mortality and Morbidity
CDIS: Child Development Information System
CS: Caesarean Section
CI: Confidence Interval
MCH: Maternal and Child Health
SEIFA: Socio-Economic Indexes for Areas
SACC: Standard Australian Classification of Countries
WHO: World Health Organisation
VPDC: Victorian Perinatal Data Collection
